# Mechanisms Linking Inflammation to Insulin Resistance

**DOI:** 10.1155/2015/508409

**Published:** 2015-06-02

**Authors:** Li Chen, Rui Chen, Hua Wang, Fengxia Liang

**Affiliations:** ^1^Hubei University of Chinese Medicine, Wuhan 430061, China; ^2^Hubei Provincial Collaborative Innovation Center of Preventive Treatment by Acupuncture and Moxibustion, Wuhan 430061, China; ^3^Integrated TCM and Western Medicine Department, Union Hospital, Tongji Medical College of Huazhong Science and Technology University, Wuhan 430022, China; ^4^Acupuncture and Moxibustion College, Hubei University of Chinese Medicine, Wuhan 430061, China

## Abstract

Obesity is now widespread around the world. Obesity-associated chronic low-grade inflammation is responsible for the decrease of insulin sensitivity, which makes obesity a major risk factor for insulin resistance and related diseases such as type 2 diabetes mellitus and metabolic syndromes. The state of low-grade inflammation is caused by overnutrition which leads to lipid accumulation in adipocytes. Obesity might increase the expression of some inflammatory cytokines and activate several signaling pathways, both of which are involved in the pathogenesis of insulin resistance by interfering with insulin signaling and action. It has been suggested that specific factors and signaling pathways are often correlated with each other; therefore, both of the fluctuation of cytokines and the status of relevant signaling pathways should be considered during studies analyzing inflammation-related insulin resistance. In this paper, we discuss how these factors and signaling pathways contribute to insulin resistance and the therapeutic promise targeting inflammation in insulin resistance based on the latest experimental studies.

## 1. Introduction

Insulin resistance (IR) is a complicated condition in which three primary metabolic tissues that are sensitive to insulin, skeletal muscle, liver, and white adipose tissue (WAT) become less sensitive to insulin and its downstream metabolic actions under normal serum glucose concentrations [1]. IR is closely associated with obesity, hypertension, hyperglycaemia, polycystic ovary syndrome, and metabolic syndrome (see glossary) [2, 3]. As the key component of metabolic syndrome, IR is also closely associated with nonalcoholic fatty liver disease (NAFLD) [4]. The antilipolytic effect of insulin is decreased in insulin-resistant conditions, which may promote hepatic triglyceride synthesis. Another feature of insulin resistance is an increasing release of free fatty acid. As we know, FFA could be taken up by organs and accumulated as ectopic fat, such as hepatic and cardiac lipids [5]. And hepatic lipids including triglyceride deposition are involved in the pathogenesis and development of NAFLD. Several factors are implicated in the pathology of obesity-related NAFLD, including complex interactions between glucose and lipid metabolism, genetic predisposition, environmental conditions, and modulation of the intestinal microbiota [6]. IR encompasses a wide spectrum of disorders, such as defective insulin receptor signal transduction and mitochondrial function [7, 8], microvascular dysfunction [9, 10], and inflammation [11–13]. Obesity, characterized as a state of chronic low-grade inflammation caused by overnutrition, is a major cause of decreased insulin sensitivity, which makes obesity a major risk factor for IR [14–16]. Obesity, also manifested as excess adiposity, is a main cause of NAFLD [17]. NAFLD is recognized as a typical feature of metabolic syndrome and manifested as a series of hepatic injuries including steatosis, nonalcoholic steatohepatitis (NASH), and even hepatocellular carcinoma [18]. Obesity causes lipid accumulation in adipocytes, which activates c-Jun N-terminal kinase (JNK) and nuclear factor-kappa B (NF-*κ*B) signaling pathways and might subsequently increase the production of proinflammatory cytokines such as tumor necrosis factor-alpha (TNF-*α*) and interleukin-6 (IL-6) [11, 19]. In most cases, adipose tissue (AT) is an important site of obesity-induced IR, and it can also affect the liver and muscle by releasing cytokines, including adipokines such as TNF-*α* [11, 18]. AT consists of several cell types. Among these, adipocytes and immune cells, such as macrophages and dendritic cells (DCs), have attracted significant attention as contributors that link inflammation to IR.

This review will focus on the relationship between inflammation and IR, and we analyze the mechanisms relating to how inflammatory cytokines, signaling pathways, and some other factors link inflammation to IR.

## 2. Cytokines That Link Inflammation to IR

### 2.1. TNF-*α*

**Figure 1:**
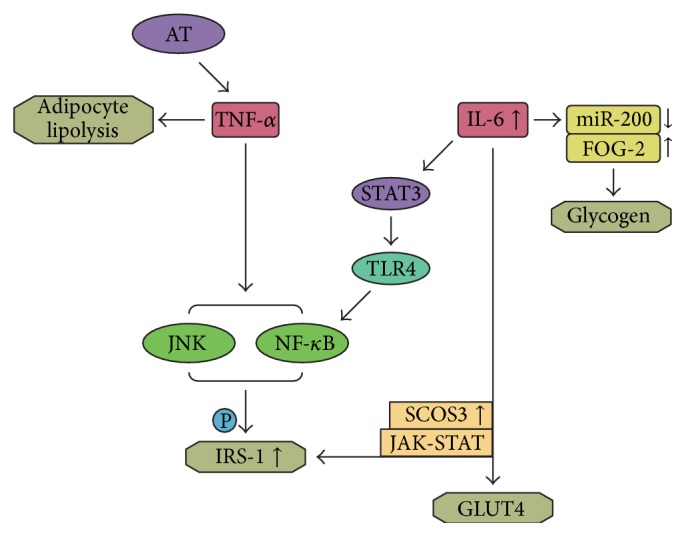
Influence of the inflammatory cytokines on the status of insulin resistance. TNF-*α* causes insulin resistance by enhancing adipocyte lipolysis stimulating JNK and IKK*β*/NF-*κ*B pathway which may increase serine/threonine phosphorylation of IRS1. IL-6 induces IR by reducing the expression of GLUT4 and IRS-1 by activating the JAK-STAT signaling pathway and increasing SOCS3 expression, and IL-6 can also lead to IR in skeletal muscle by inducing TLR-4 gene expression through activation of STAT3; besides, TLR4 is suggested to be major upstream molecules in the activation of NF-*κ*B. Besides, IL-6 is also found to induce IR by impairing the synthesis of glycogen through downregulating the expression of miR-200s and upregulating that of FOG-2.

Studies of TNF-*α* in the 1990s first analyzed the relationship between inflammation and IR [20]. TNF-*α* is an adipose tissue-derived proinflammatory cytokine that causes insulin resistance by enhancing adipocyte lipolysis and increasing the serine/threonine phosphorylation of IRS-1 (insulin receptor substrate-1) [11, 21]. Several signaling pathways, including the IKK*β*/NF-*κ*B pathway, are involved in the pathogenesis of IR (see Figure 1) [22, 23]. It was reported that TNF-*α* can increase glucose uptake in both visceral and subcutaneous adipocytes by activating the adenosine monophosphate activated protein kinase (AMPK) pathway, whereas it triggers insulin resistance in visceral adipocytes by activating JNK1/2. Because of the depot-specific effects of TNF-*α* on glucose uptake, approaches to treat IR by modulating TNF-*α* signaling are ongoing [24]. However, studies of therapies such as the TNF-*α* superfamily member sTWEAK (soluble tumour necrosis factor-like weak inducer of apoptosis), which aims to block TNF signaling to treat IR, have demonstrated that TNF-*α* plays a role in IR [25]. Interestingly, the plasma levels of TNF-*α* are higher in males than in females, as well as in obese individuals compared with lean ones. This suggests that obese males are more likely to suffer from IR and related diseases such as cardiovascular disease [26].

### 2.2. IL-1*β*

Interleukin-1*β* (IL-1*β*) is a proinflammatory cytokine whose secretion is regulated by inflammasome activity. IL-1*β* contributes to IR by impairing insulin signaling in peripheral tissues and macrophages, which leads to the reduced insulin sensitivity of *β*-cells and possible impaired insulin secretion [27, 28]. The levels of IL-1*β* in various cells such as endothelial cells and monocytes are increased during hyperglycemia [29]. IL-1*β* also plays a vital role in initiating and maintaining inflammation-induced organ dysfunction in type 2 diabetes mellitus (T2DM) [30]. IL-1*β* might increase systemic inflammation and inhibit insulin action in the major insulin-target cells, such as macrophages [31].

### 2.3. IL-6

IL-6 is secreted by multiple tissues, particularly adipose tissue, and is recognized as an inflammatory mediator that causes IR by reducing the expression of glucose transporter-4 (GLUT-4) and insulin receptor substrate-1 (IRS-1). These effects are exerted by the activation of the Janus kinase-signal transducer and activator of transcription (JAK-STAT) signaling pathway (see Box 1) and increased the expression of suppressor of cytokine signaling 3 (SOCS3) [32, 33] (see Figure 1). Therefore, hybrid training can ameliorate insulin resistance by suppressing serum IL-6 in skeletal muscle [34]. IL-6 also induces IR by blocking the phosphoinositide 3-kinase (PI3K) pathway and impairing glycogen synthesis by downregulating the expression of microRNA-200s (miR-200s) and upregulating that of friend of GATA 2 (FOG-2) [35, 36]. It was suggested that IR in human skeletal muscle is related to IL-6 stimulation, which induces toll-like receptor-4 (TLR-4) gene expression by activating STAT3 [37] (see Figure 1).

### 2.4. Leptin

Leptin is a protein that is derived primarily from white adipose tissue (WAT) [38]. It suppresses appetite and increases energy expenditure by repressing anabolic neuronal circuits and activating catabolic neuronal circuits. In addition, leptin levels are affected by nutriture [39]. Leptin-mediated appetite and energy homeostasis are associated with the progression of IR [40]. Furthermore, a state called leptin resistance, which was disputed lately by the concept of hypothalamic leptin insufficiency, is often observed in the obese individuals, and weight loss simultaneously reduces serum leptin levels. This suggests that leptin might have a role in regulating IR. Consistent with this, the stimulation of PI3K signaling by leptin is essential for modulating glucose metabolism and the function of pancreatic *β*-cells [31–42]. It is likely that an increased concentration of leptin, an anti-inflammatory cytokine, during inflammation in AT is associated with leptin resistance in obese individuals. Interestingly, leptin was recommended as a biomarker for* in utero* insulin resistance based on the link between maternal and fetal leptin and IR [43, 44]. Leptin is a potential treatment for IR because it improves glycometabolism, insulin sensitivity, and lipometabolism [45, 46].

### 2.5. Adiponectin

Adiponectin is produced mainly by WAT. Its levels reduce in obesity, IR, or T2DM, where it acts as an anti-inflammatory cytokine, but increase in osteoarthritis (OA) and type 1 diabetes mellitus (T1DM), where it acts as a proinflammatory cytokine [39, 47]. Two receptors are involved in the glucose metabolism that links adiponectin to the amelioration of IR. Adiponectin receptor 1 (AdipoR1) is likely to reduce the expression of the genes that encode hepatic gluconeogenic enzymes and molecules involved in lipogenesis by activating AMPK. In contrast, adiponectin receptor 2 (AdipoR2) increases the expression of the genes that contribute to glucose consumption by activating peroxisome proliferator activated receptor-alpha (PPAR-*α*) signaling [48, 49]. AdipoR1 and AdipoR2 are expressed at high levels in skeletal muscle and the liver, respectively [28, 50]. In brief, adiponectin ameliorates hepatic insulin resistance by reducing glycogenesis and lipogenesis, as well as increasing glucose consumption.

### 2.6. Resistin

The production of resistin is complex. In rodents, it is generated from adipocytes, whereas it is produced mostly by macrophages in humans. Its concentrations increase concurrently with the levels of inflammatory mediators [51]. It was suggested that resistin participates in the pathogenesis of IR and that its levels might be elevated due to obesity and IR [52]. Resistin promotes IR by regulating the expression of proinflammatory cytokines, including TNF-*α* and IL-6, in macrophages via an NF-*κ*B-dependent pathway. It also plays roles in inflammation and IR by binding directly to TLR4 receptors in the hypothalamus to activate JNK and mitogen-activated protein kinase (MAPK) signaling pathways [53].

### 2.7. MCP-1

Monocyte chemoattractant protein-1 (MCP-1) is a proinflammatory chemokine produced by adipocytes, macrophages, and endothelial cells, which might lead to the recruitment of macrophages, DCs, and memory T cells [11, 54]. Adipocytes and macrophages are the main source of proinflammatory cytokines. However, the expression of MCP-1 increases during adiposity, which might stimulate the recruitment of macrophages and DCs, which further increases the expression of cytokines to exacerbate inflammation-induced IR [22]. The expression of MCP-1 increases during obesity, particularly in visceral fat areas, which might contribute to the pathogenesis of IR, particularly in the liver [54, 55]. It plays a role in IR by regulating the inflammatory response, insulin sensitivity, lipid metabolism, macrophage polarization and infiltration, and the phosphorylation of extracellular signal-regulated kinase-1/2 (ERK-1/2) and p38 MAPK [56]. C-C motif chemokine receptor 2 (CCR2) is a vital MCP-1 receptor. In adipose tissue of CCR2 knockout mice, macrophage content and inflammatory profile were reduced. CCR2 deficiency also ameliorated hepatic steatosis and improved insulin sensitivity [57]. This suggests that MCP-1 plays a crucial role in the development of both inflammation and IR.

## 3. Signaling Pathways Linking Inflammation to Insulin Resistance

### 3.1. IKK*β*/NF-*κ*B Pathway

**Figure 2:**
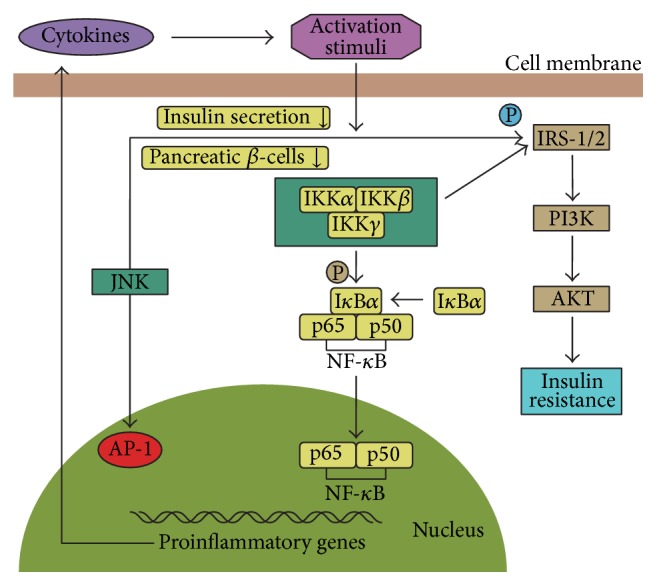
Inflammatory pathways linking inflammation to insulin resistance. Activation of JNK and NF-*κ*B pathways causes serine kinase phosphorylation of IRS-1 or IRS-2, which may block insulin signaling and finally lead to the occurrence of IR. In addition, JNK and NF-*κ*B pathways are involved in the production of proinflammatory cytokines which may in turn become activation stimuli of the pathways.

NF-*κ*B is a transcription factor comprised of Rel family proteins such as p65/RelA, RelB, c-Rel, p50/p105, and p52/p100. It is involved in a series of pathological processes such as inflammation and innate and adaptive immune responses [58, 59]. NF-*κ*B is sequestered in the cytoplasm bound to I*κ*B proteins in normal circumstances, which prevents the nuclear localization of NF-*κ*B. After stimulation with various pathogenic stimuli, such as those in obese individuals, the IKK complex that contains two subunits (IKK*α* and IKK*β*) is activated, which triggers the phosphorylation of I*κ*B*α* on Ser32 and 36. This leads to the degradation of I*κ*B*α*, exposes the nuclear localization sequence of NF-*κ*B, and triggers its translocation to the nucleus and the upregulation of target genes that encode inflammatory mediators such as TNF-*α*, IL-1*β*, and IL-6 [20, 58] (see Figure 2). IKK*β* deficiency in adipocytes completely prevented the free fatty acid- (FFA-) induced expression of TNF-*α* and IL-6, whereas the activation of IKK*β* inhibited the expression of anti-inflammatory cytokines such as leptin and adiponectin [60]. According to this, the deletion of IKK*β* improved glucose tolerance and insulin sensitivity [61]. In addition, treatments that inhibit NF-*κ*B always improve IR, which suggests that the NF-*κ*B pathway plays an important role in inflammation-associated IR [62]. NF-*κ*B is also a vital intermediary that couples IR to the proinflammatory cytokine IL-1*β* in IR-related diseases such as obesity and T2DM [27].

### 3.2. JNK Pathway

There are three different JNK isoforms (JNK-1, -2, and -3), which belong to MAPK family. JNK contributes to inflammation and metabolic syndrome (MS), obesity, and IR by regulating the production of proinflammatory cytokines, karyomitosis, and cellular apoptosis [63–65]. JNK can also be stimulated by endoplasmic reticulum (ER) stress, which leads to the serine phosphorylation of IRS-1 (see Figure 2). JNK plays a role in the phosphorylation of the c-Jun component of activator protein (AP-1) transcription factor, but there is no evidence of a direct relationship between this transcriptional pathway and JNK-reduced IR. The JNK pathway can be activated under diabetic conditions, which might increase IR. Conversely, suppressing the JNK pathway improves IR and glucose tolerance [66]. JNK plays an important role in IR by inhibiting insulin secretion from pancreatic *β*-cells via proinflammatory stimuli including IL-1. Moreover, the excessive activation of JNK in peripheral insulin-sensitive tissues promotes IR [67]. It was demonstrated that inhibiting JNK reduced the release of IR-related proinflammatory cytokines such as TNF-*α* and MCP-1 [68–70]. Interestingly, JNK-1 deficiency in adipose tissue protects against hepatic steatosis and promotes glucose intolerance, insulin clearance, IR, and hepatic steatosis. In skeletal muscle, JNK-1 does not affect the development of obesity and IR [65, 71]. However, JNK in isolated rat skeletal muscle plays a vital role during oxidant-induced IR because insulin-stimulated glucose transport activity was improved by the selective inhibition of JNK [72]. Taken together, these studies suggest that further studies are needed to analyze the effects of JNK in IR.

### 3.3. Inflammasome Pathway

**Figure 3:**
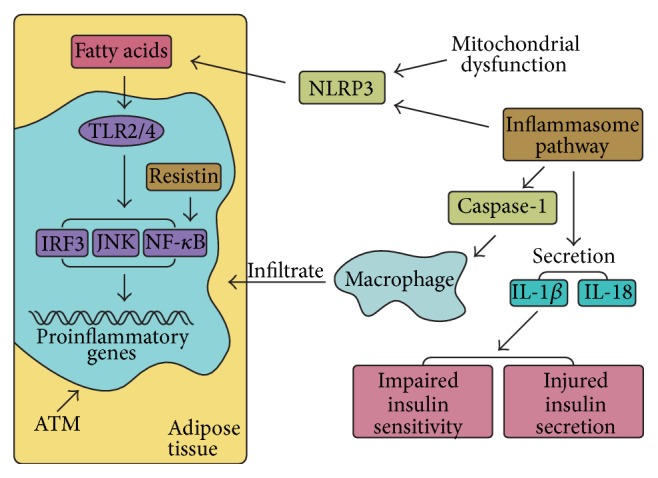
Inflammasome pathway and macrophages are involved in development of insulin resistance. The secretion of IL-*β* and IL-18 can be regulated by inflammasome pathway. Inflammasome consists of a large group of cytosolic protein complexes including NLRP3 and caspase-1. NLRP3 can be activated by mitochondrial dysfunction through causing ROS accumulation, and NLRP3 is also a novel molecular link between saturated FFA and chronic inflammation. Caspase-1 mediates macrophages that infiltrate into adipose tissues. Dietary saturated fatty acids lead to activation of TLR2 and TLR4 in ATMs, giving rise to the activation of IRF3, JNK, and NF-*κ*B.

The inflammasome consists of a large group of cytosolic protein complexes and plays roles in inflammation by regulating the secretion of IL-1*β* and IL-18. Therefore, it is important in innate immunity and metabolic syndromes such as obesity and IR [30, 73]. NOD-like receptor proteins (NLRPs), neutrophilic alkaline phosphatases (NALPs), apoptosis associated speck-like protein (ASC), and caspase-1 are the essential components of inflammasome complexes [20]. Inflammasome NLRP3 (nucleotide-binding domain, leucine-rich-containing family, and pyrin domain-containing-3), which links saturated FFAs to chronic inflammation, is being studied extensively because it is highly sensitive to nonmicrobial stress. It can be activated by mitochondrial dysfunction. In addition, the reduced expression of NLRP3 in obesity results in enhanced insulin signaling, decreased inflammation, and improved insulin sensitivity [73, 74] (see Figure 3). Caspase-1 is a cysteine protease that contributes to IR by counteracting the metabolic function of adipose tissue to impair insulin sensitivity and also mediates the infiltration of macrophages into adipose tissues [75, 76]. It was reported that the elimination of ASC and caspase-1 lowers the plasma levels of insulin, leptin, and resistin. Moreover, ASC deficiency might protect individuals against HFD-induced IR, hepatic steatosis, and adipocyte hypertrophy. In addition, caspase-1-deficient mice have high energy expenditure. Taken together, these studies suggest that the inflammasome plays a vital role in obesity-induced IR and that it is an important therapeutic target for the treatment of IR [76].

## 4. Other Factors Linking Inflammation to IR

### 4.1. Macrophages

Macrophages infiltrate and reside in adipose tissue, named ATMs, and usually play an important role in obesity-induced IR. There are two types of ATM: classically activated (M1) in obese animals and alternatively activated (M2) in lean species [77]. ATMs have an important role in the development of chronic inflammation, including obesity-induced inflammation, because they are the primary source of cytokine production. In addition, obesity might change the number of ATMs by increasing the triple positive CD11b + F4/80 + CD11 + ATM subpopulation [20]. As well as using CD11c as an M1 marker, Fujisaka et al. used CD206 rather than CD209 and CD301 as M2 markers by flow cytometry to demonstrate that IR might be regulated by the number of M1 ATMs and the M1 : M2 ratio. In addition, intervention with pioglitazone could reduce inflammation and ameliorate IR by upregulating the expression of IL-10, which might contribute to the reduction of M2 quantity [77]. In another study, it was suggested that the MCP-1/CCR2 axis might contribute to a shift from M2 to M1 polarization, which is an important cause of IR as it leads to the production of inflammatory factors such as TNF-*α* and IL-6 [78]. During obesity, dietary saturated fatty acids lead to the activation of TLR2 and TLR4 in ATMs, which is followed by the activation of interferon regulatory factor-3 (IRF3), JNK, and NF-*κ*B and subsequent inflammatory signaling (see Figure 3) [1].

### 4.2. hs-CRP

C-reactive protein (CRP) is an acute-phase protein synthesized by the liver. It is an inflammatory marker whose expression is increased significantly during inflammation, mainly due to its regulation by proinflammatory cytokines such as IL-6 and TNF-*α* [79, 80]. In most clinical and scientific studies, CRP is measured using high-sensitivity assays and is known as high-sensitivity CRP (hs-CRP) [81]. It was suggested that increased hs-CRP levels might be caused by an insufficient insulin-induced suppression of CRP synthesis. Moreover, CRP might contribute to vascular inflammation by activating complement proteins and increasing the production of thrombogenic components bound to the membranes of injured vascular cells, which contributes to the development of IR [80]. In addition, elevated CRP expression is a potential risk factor and indicator for T2DM. However, there is no apparent causality between serum CRP, IR, and diabetes, which suggests that CRP is more likely to be a downstream marker rather than an upstream effector that links inflammation to IR [82]. Nevertheless, hs-CRP is closely associated with IR, and thus its expression should be assessed during investigations of IR.

## 5. Concluding Remarks

Inflammation plays an important role in the development of IR via various cytokines and molecular pathways, and so inflammation should be targeted with appropriate interventions to prevent IR. Because dietary fat might play a role in the production of inflammatory molecules by modifying the intestinal microbiota, which might result in an inappropriate immune reaction [83], it is important for individuals to develop good dietary and living habits. Specific factors and signaling pathways are often correlated with each other. For example, the activation of IKK*β*/NF-*κ*B signaling might increase the secretion of proinflammatory cytokines such as TNF-*α* and IL-1*β*, which might in turn stimulate IKK*β*/NF-*κ*B signaling. Therefore, both of the fluctuation of cytokines and the status of relevant signaling pathways should be taken into account during studies analyzing inflammation-related IR. Most current studies of inflammation-related IR are performed in animals, which makes it challenging to apply these methods to humans to exert a curative effect of IR in the clinic. Because the mechanisms that link inflammation to IR are not understood completely, additional well-designed clinical and laboratory studies are in demand to elaborate their relationship.

## 6. Box 1

### 6.1. The JAK-STAT Signaling Pathway

The Janus kinase-signal transducers and activators of transcription (JAK-STAT) signaling pathway are a cytokines-activated cascade involved in many important biological processes including the proliferation, differentiation, and apoptosis of the cells [84]. This signaling pathway contains three components: tyrosine kinase associated receptor, Janus kinase and signal transducer, and activator of transcription [85]. To date, four members of JAK kinase family have been identified including JAK1, JAK2, JAK3, and TYK2, and the STAT family consists of seven proteins (STATs 1, 2, 3, 4, 5A, 5B, and 6) [86]. The signaling pathway is initiated through binding of ligands to membrane-bound receptors which may lead to receptor dimerization and then activate the JAK kinases; in turn, the activation of JAK kinases phosphorylates the tyrosine residues with the receptors [87]. As a result, STAT proteins are phosphorylated by JAK, then dimerize via their src-homology 2 (SH2) domains, and translocate to the nucleus where they regulate transcription of specific target genes involved in multiple diseases including leukemia, rheumatoid arthritis, cancer, and diabetic nephropathy [88, 89].

## Glossary

*Metabolic Syndrome.* A pathophysiological disorder characterized by a cluster of risk factors for cardiovascular disease, type 2 diabetes, and renal disease.

*Adenosine Monophosphate Activated Protein Kinase.* A key molecule implicated in metabolic modulation as it increases O_2_ consumption, glucose metabolism, and fatty acid oxidation.

*Soluble Tumour Necrosis Factor-Like Weak Inducer of Apoptosis.* Tumor necrosis factor-like weak inducer of apoptosis (TWEAK) is a member of the tumor necrosis factor (TNF) superfamily. Soluble tumour necrosis factor-like weak inducer of apoptosis (sTWEAK) is a soluble variant of TWEAK. sTWEAK plays a role in a series of biological processes including cellular proliferation, differentiation, apoptosis, and inflammation.

*Glucose Transporter.* It is a wide group of membrane proteins that facilitate the transport of glucose.

*Phosphoinositide 3-Kinase.* It is an enzyme that generates lipid second messenger molecules, resulting in the activation of multiple intracellular signalling cascades.

*Friend of GATA.* The GATA family refers to a kind of transcription factors that recognizes and binds to the GATA motifs. GATA proteins play an essential role in hematopoiesis and tissue specific gene expression through functional interactions with friend of GATA (FOG-) proteins. There are two FOG proteins, FOG-1 and FOG-2. FOG-1 is mainly expressed in hematopoietic tissues and FOG-2 in the heart, brain, and gonads.

*Toll-Like Receptors.* Toll-like receptors are pattern recognition receptors that play an important role in recognizing the conserved molecular structure of pathogens and triggering of the innate immune response.

*Peroxisome Proliferator Activated Receptors.* Peroxisome proliferator activated receptors are a group of ligand-activated nuclear receptors involved in the gene expressions associated with the metabolic processes.

*Mitogen-Activated Protein Kinase.* It is an important signal transducer acting as a regulator of physiology and immune responses.
